# Ant *Lasius niger* joining one-way trails go against the flow

**DOI:** 10.1038/s41598-022-05879-4

**Published:** 2022-02-11

**Authors:** Yuta Sakamoto, Tomoko Sakiyama

**Affiliations:** Department of Information Systems Science, Faculty of Science and Engineering, Soka University, 1 Chome-236 Tangimachi, Hachioji, Tokyo 192-8577 Japan

**Keywords:** Computational biology and bioinformatics, Ecology

## Abstract

Social insects, such as ants, use various pheromones as their social signal. In addition, they use the presence of other ants for decision-making. In this study, we attempted to evaluate if individual decision-making is influenced by the complementary use of pheromones and presence of other ants. Ants were induced to form a one-way flow system. We found that when ants entered such a system at a right angle, they tended to move in the opposite direction of the one-way flow system. Interestingly, the target ants moved randomly in the experiments in which no ant and/or no pheromone trails were present. We also developed simulation algorithms and found that artificial ant foragers could reach a certain goal more often if they adopted the reverse run (similar mechanism found in ant experiments) over the forward run (moving in the same direction as their nestmates).

## Introduction

Chemical communications of ant foragers are well known as one of the self-organization phenomena. Ant foragers usually deposit pheromones when returning to their nest after finding a food item. Pheromones act as signals and recruit other ants to food resource^1–6^. These other ants also deposit pheromones, resulting in the establishment of a pheromone trail^6,7^. According to previous research, argentine ant foragers modify their travel direction by reacting to the gradient of pheromone concentrations, suggesting that ants continuously update their travel direction by scanning pheromone concentrations around them^5^. In fact, they may consider other factors in addition to the pheromone trail, such as geometrical information, route memory, and the presence of other ants, which may result in the selection of a path among multiple paths^8–11^ In fact, ant workers modulate trail following, which may depend on factors such as visual information^12^. In summary, ants do not always obey pheromones blindly.

Several studies have reported that the presence of nestmates on the pheromone trail coordinates the actions of ant foragers^8,13,14^. Black garden ants *Lasius niger* decrease the rates of pheromone depositions when they encounter their nestmates on the trail and at the food source, suggesting that ant foragers may modulate the overpopulation on a foraging route based on physical contact with the nestmates^13,15^. However, it is also reported that foraging ant groups tend to select a pheromone trail occupied by their nestmates^8^. Thus, it is possible for ant foragers to respond differently to the information, which may depend on the presence of ant nestmates. Moreover, physical contact with the nestmates may also determine the foraging action of ant workers^9,16,17^. Considering the abovementioned information, the movement of the garden ant *L. niger*, particularly naïve ants, on the trail may be influenced by the encounter with ant nestmates. This is because experienced ants will confidently move forward on a trail owing to their own experiences^18^. Contrarily, naïve ants may have to use additional information to update their position on a trail. A similar mechanism was observed in laden *Pheidologeton diversus* workers on a foraging trail. They used the flow of other laden workers to navigate themselves toward the nest^17^, suggesting that ant workers tended to follow a stream of ant traffic.

Few studies have focused on the orientation cue on a pheromone trail at individual-level and its relation with the presence of other ants although several researches have reported that *L. niger* workers could modify their actions on a pheromone trail, as already mentioned. Here we tackled this issue and investigated a possibility that contacting other ants oriented *L. niger* workers on a pheromone trail toward a certain direction. Consequently, we investigated the following question in this study: which mechanism do *L. niger* naïve workers rely on? To be more precise, we assessed whether they move in the same direction with ant nestmates or in the opposite direction from ant nestmates when coming in contact with them on the trail. To investigate this, we developed a maze apparatus with the following two features: (1) A set-up to establish one-way ant trails. By doing so, we could separate the outbound trips of ant foragers from their inbound trips^19^. (2) Individual naïve ants were allowed to join the ant traffic at right angles to a pheromone trail to easily judge whether they choose both sides (right and left side for those ants) equally if they do not modify their movements when coming in contact with ant nestmates on the trail^20^. Herein, we propose that the garden ant *L. niger* uses two information sources—pheromones and presence of other ants—to navigate through the established ant trail when entering the trail at a right angle.

Using this maze apparatus, we hypothesized that individual ants, which entered the established ant trail at a right angle, followed the same direction of other nestmates using two complementary information sources—pheromones and presence of other ants. Contrary to our expectation, however we found that ants tended to move in the opposite direction of the one-way flow system. We also developed a multiagent-based model to evaluate the mechanistic understanding of the action of individual ants.

## Methods

### Ant experiments

#### Ant colonies

Seven colonies of the garden ant *L. niger* collected from the *Soka University* and a nearby park were used in this study (Extended Data Table S1). They were placed in plastic cases (35 × 25 × 6 cm). Water was provided ad libitum. They were fed a sucrose solution, and were starved for 2–5 days before the start of the experiment. The colonies were queen-less colonies with 200–700 workers. Aqueous sucrose solution was used as a food resource (bait) in the experiments. The laboratory room where the experiments were performed and the ant colonies were kept was maintained at a temperature of 25–27 °C and a humidity of 60–70%. Artificial lights were also installed in this room.

#### Apparatus

**Figure 1:**
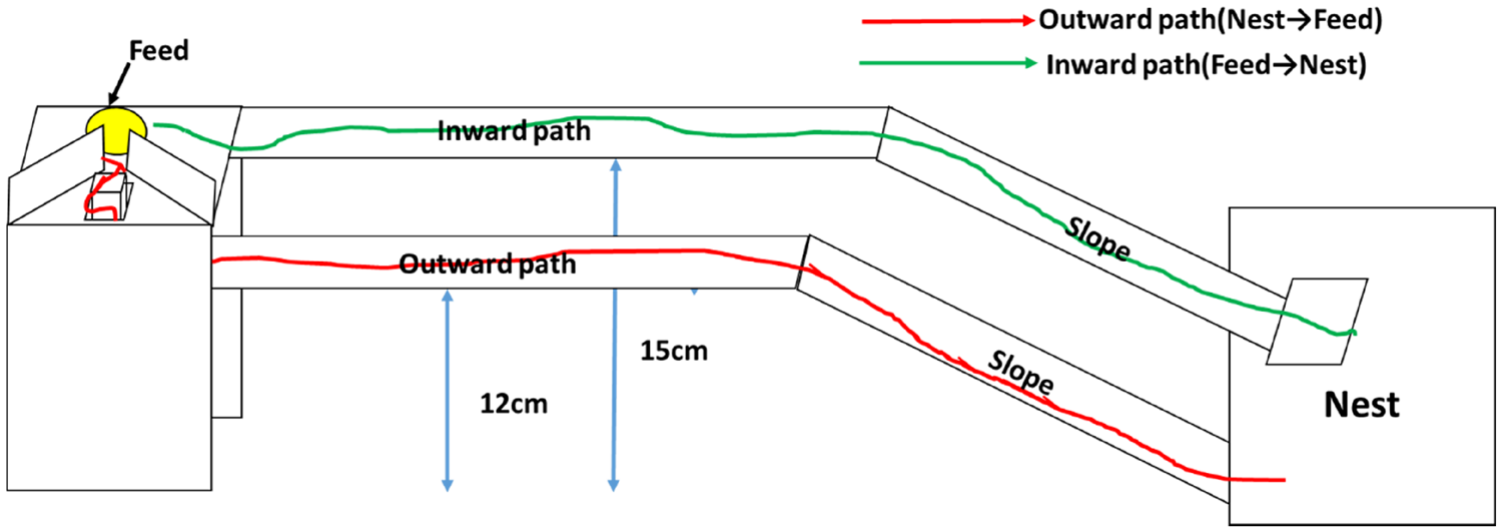
The main apparatus used in the three experiments (the main experiment and the comparison experiments 1 and 2). Nests are connected to the experimental apparatus by a slope. In the main experiment, on the outward path, there is ant traffic from the nest to the feeding site on a pheromone trail, and on the inward path, there is ant traffic from the feeding site to the nest on a pheromone trail. In the comparison experiment 1, only a pheromone trail is present on both the outward and inward paths. In the comparison experiment 2, no pheromone trail or ant traffic is present on both the outward and inward paths.

We used an apparatus, called “the main apparatus,” with two paths from the nest to the feeding site (length: 30 cm, width: 2 cm, height: 12 cm for the outward path and 15 cm for the return path) (Fig. 1). This apparatus could separate the outward path (bridge) from the inward path (bridge). Here, the outward path refers to that taken by ants from the nest to the feeding site, whereas the inward path refers to the path taken by ants from the feeding site to the nest.

Two important features of this apparatus were as follows: firstly, it allowed ants to only enter the outward path from the nest. A rat-guard structure at the end of the inward path prevented the ants on the outward path from entering the inward path (Extended Data Fig. S1A). Secondly, we installed a vertical structure at the end of the outward path (height: 4 cm). After climbing the vertical structure, ants were not allowed to return to the outward path (Extended Data Fig. S1B). Moreover, we installed partitions on the feeding site, which also prevented ants from returning to the outward path after reaching the feeding site (Extended Data Fig. S1C). After entering the feeding site, ants had to pass through a narrow gap (width: 0.5 cm) created by the partition. No visual cues were offered as the apparatus was surrounded on all four sides by plastic walls.

In this experiment, we made another apparatus for a single ant (target ant), which would be joining the ant trail on the main bridges (Extended Data Fig. S2). This apparatus, called “the confluence device,” was a detachable device that could be connected at right angles to the outward and inward bridges of the main apparatus. To connect this device to the outward bridge, we made the confluence path of this device under the inward bridge of the main apparatus, since the outward bridge was lower than the inward bridge. Thus, we made a slope on the outward confluence path connected to the outward bridge of the main apparatus. Further, because placing the ants directly on the sidewalk sometimes caused them to fall off the sidewalk owing to panic, we constructed a free space and a wall (height: 5 cm) in the middle of the confluence device on which the ants were placed calmly. Owing to this modification, we could let each target ant calm down and then access the main bridge whenever they wanted to. The apparatus used in this experiment was made of white plastic plates.

#### Pheromone trail with ant traffic

**Figure 2:**
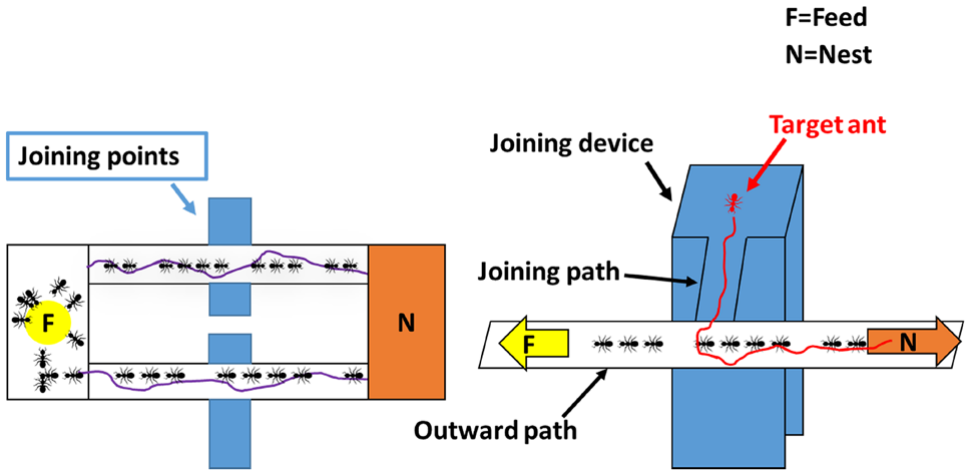
Four joining points (LL, LR, RL, and RR) and the confluence device (joining device). The confluence (joining) device was connected at right angles to the center of the outward and inward bridges of the main apparatus. Here, the LR version is shown as an example.

This main experiment was limited to once a day for each colony. A sucrose solution was dripped into the feeding site. Target ants, which were walking on a plastic case as foragers, had been moved from their nests to another case immediately before a trail of (nontarget) ants was formed. Thus, dozens of ants were moved in advance to the case to be used as target ants. Subsequently, a trail of (nontarget) ants was formed from the nest to the main apparatus. Considering that it took some time for the ants that had finished foraging and returned to the nest to recruit their mates, the ants were left for approximately 40 min to an hour until a permanent ant trail was formed. It was difficult to form an ant trail immediately after the start of the experiment since no foraging pheromones could be produced in the first foraging trip on the outward path and since experienced foraging ants may make foraging pheromones on the outward path^2,21,22^. The target ants were allowed to enter bridges of the main apparatus after the establishment of a permanent ant trail. At that time, trails of individual target ants were started. Target ants were allowed to join at right angles to the path on the apparatus, one by one from the confluence device. Individual target ants were allowed to enter the main apparatus at four different points: (1) Left-Left (LL), located at the left side of the center of the outward path. The outward path was on the left side, whereas the inward path was on the right side for the experimenter when seen from the nest. (2) Left–Right (LR), located at the right side of the center of the outward path. (3) and (4) Right-Left (RL) and Right-Right (RR), located at the left and right sides of the center of the inward path, respectively (Fig. 2). We had set these four points to check if target ants tended to turn their body to a certain direction when entering the main bridges, regardless of the movement direction of the other ants. A video camera (Panasonic, AVCHD 30fps) was used to record the migration of ants to the feeding site or nest. Videos were taken from above, and target ants were used only once.

The goal lines were set at 15 cm from the center of the main paths. We checked the side (nest side or feeding site side) from which a target ant passed the goal line.

#### Pheromone trail with no ant traffic

This comparison experiment 1 was limited to once a day for each colony. Dozens of ants were moved in advance to another case to be used as target ants in a similar manner to the main experiment. The (nontarget) ants were left for about 40 min to an hour until a permanent ant trail was formed. Subsequently, we removed all the ants from the device. Then, target ants were allowed to enter on the side path one by one. In this case, we left the bait in place to control this experiment under the same condition as the main experiment. As the pheromone trail was created on the outward path as well as on the inward path, it was the only decision-making factor for the ants to join at the main path (outward/inward paths). We checked the side (nest side or feeding site side) from which a target ant passed the goal line in a similar manner to the main experiment.

#### No pheromone trail or ant traffic

This comparison experiment 2 was limited to once a day for each colony. Dozens of ants were moved in advance to another case to be used as target ants in a similar manner to the main experiment. This experiment was conducted to investigate ant behavior under the following two conditions: (1) no ant trails and (2) no pheromones trails. The bait was in place in the same manner. We checked the side (nest side or feeding site side) from which a target ant passed the goal line in a similar manner to the main experiment. After each trial (the target ant passed the goal line), we wiped the apparatus with ethanol solution before the next target ant was allowed to enter the main paths.

#### Analysis

The goal lines were set at 15 cm from the center of the main paths. We checked which goal side the target ants reached the goal line on each trial. A reverse run referred to the goal to the nest on the outward path and the goal to the feeding site on the inward path. A normal run referred to the goal to the feeding site on the outward path and the goal to the nest on the inward path.

In some cases of the main experiment, foraging (nontarget) ants that could not reach the feeding site on their outward path or could not return to the nest on their inward path would be against the ant flows. On the outward path, we considered that the ants conducted a “reverse flow” if the position of their heads was on the nest side compared with the position of their stomach. If not, we defined that the ants conducted a “normal flow” (Extended Data Fig. S3). On the inward path, we defined that the ants conducted a “reverse flow” if the position of their head was on the feeding site side compared with the position of their stomach. If not, we defined that the ants conducted a “normal flow” (Extended Data Fig. S3). We focused on the target ants that came in contact with ants with normal flow. Therefore, if an ant with reverse flow was located within 10 cm of the target ant, that trial was excluded from the analysis.

Furthermore, we also evaluated if target ants coming in contact with foraging (nontarget) ants immediately after entering the trail would affect the goal choice. Therefore, we conducted an analysis focusing on the contact using the data from the main experiment. We examined whether or not the target ant made contact with other foraging ants until it passed a point 2 cm from the center of the path. As already mentioned, if the target ant came in contact with another ant moving against the normal flow of the ant trail, this contact was excluded from the counts. Moreover, we also excluded cases in which the body of target ants was on a point 2 cm from the center of the path by visual evaluation. Thus, we examined the goal choice of target ants by focusing on whether or not they came in contact with other ants immediately after joining the main bridges.

We also conducted a preliminary experiment using a single path apparatus to investigate bi-directional trail behaviour. Please see the Extended Data File S1.

### Model description

The models were coded using the C programming language. The model description follows the Overview, Design concepts, and Details protocol^23,24^.

#### Purpose

The purpose of the model was to examine the mechanistic understanding of our findings. We adopted an action of target agents obtained from our ant experiments and compared it with another action of target agents on a trail that was contrary to the fact. To be more precise, target agents were allowed to obey an alignment rule in which they tended to move in the same direction with other agents. We named the former model as the reverse-rule model and the latter model as the alignment-rule model. By doing so, we could find the significance of our findings from ant experiments.

#### Entities, state variables, and scales

We developed two different models (reverse-rule model and alignment-rule model) that included two types of entities: agents and cells. The agent has the state variable *Navigational state*, which has two values: *Navigational state* = {wandering, foraging}. The cell has the state variable *Pheromone*; this value represents the amount of pheromones in each cell. We used a 2D lattice field and set a straight bridge with 61 cells × 5 cell sizes. We also set goal lines at *x*-coordinate =  − 30 and 30. If the agents reached coordinates satisfying their *x*-coordinate =  − 30 or 30, they were removed from the system. If the agents reached *y*-axis boundaries, their movement direction was restricted. Each trial continued until the target agent reached one of the two goal lines. However, trials were forcibly finished if the target agent never reached any goal line by *t* = 500-time steps. In total, we conducted 1000 trials.

#### Process overview and scheduling

At the beginning of each trial, an artificial target ant (*Navigational state* = wandering) was introduced at the center of an artificial simulation field. Foraging agents (*Navigational state* = foraging) were randomly distributed on the simulation field in advance.

Agents on the simulation field selected one direction from two directions (+ *x* and − *x*) on each time step and updated their positions. Briefly, an agent at coordinate (*x*, *y*) selected one direction from two directions (+ *x* and − *x*) and updated its position with one of the three coordinates—(*x* − 1, *y*), (*x* − 1, *y* + 1), or (*x* − 1, *y* − 1)—if it selected the − *x* direction, or—(*x* + 1, *y*), (*x* + 1, *y* + 1), or (*x* + 1, *y* − 1)—if it selected the + *x* direction by scanning pheromones on these three coordinates. For example, if an agent at coordinate (0, 2) decided to move in + *x* direction at one time, the position of this agent was replaced with one of (1, 3), (1, 2) and (1, 1) from (0, 2) by scanning pheromones on these three coordinates. The target agent selected the − *x*/ + *x* direction with equal probability on each time step until it met the foragers. In contrast, foraging agents tended to decide to move in the − *x* direction on each time step with a high probability and therefore they tended to select the − *x* direction for position updating. Foraging agents deposited pheromones before leaving the current cell (see submodel entitled “Position updating” and submodel entitled “Pheromone updating”). In contrast, the target agents did not deposit pheromones.

Using above submodels, artificial ants sometimes met other agents. If the target agent (*Navigational state* = wandering) met the foragers (*Navigational state* = foraging), the target agent tended to select one direction from two directions (+ *x* and − *x*) on each time step thereafter with a high probability, which was dependent on which direction the met foragers came from. More strictly, in the reverse-rule model, the target agent tended to move in an opposite direction from the foragers if it met the foragers coming from the opposite direction. On the contrary, the target agent in the alignment-rule model tended to move in the same direction with foragers if it met the foragers moving in the same direction (see submodel entitled “The interaction between the target agent and foragers”). For example, in the reverse-rule model, if the target agent at coordinate (*x*, *y*), whose previous coordinate was (*x* − 1, *y*), met the forager coming from the opposite direction, whose previous coordinate was (*x* + 1, *y*), the target agent decided to move in + *x* direction on each time step thereafter with a high probability until similar events occurred.

#### Design concept

The mean goal time was the emergent property of the model. Sensing was important as the agents scanned the pheromone concentrations. Stochasticity was used to determine in which direction the agent moved and to select one cell using the pheromone concentrations.

#### Initialization

We set a single agent (target agent) on the coordinate (0, 2) and its *Navigational state* was set to wandering (Extended Data Fig. S5A). We also set *N* foraging agents on the bridge whose *Navigational state* was set to foraging. Therefore, *N* + 1 agents were on the test field at the beginning of each trial. A target agent was the agent *k* = 0, whereas foraging agents were agents *k* = 1, 2, …, *N*. These foragers were randomly distributed on the bridge. Thus, *x*(*k*) $\in$ {*n* |− 30 ≤ *n* ≤ 30, *n* is an integer} and *y*(*k*) $\in$ {*n* | 0 ≤ *n* ≤ 4, *n* is an integer} for *k* > 0.

Foraging agents were set to move in the -*x* direction (*Direction*(*k*) for *k* > 0 = − *x*). On the other hand, the target agent randomly chose one direction from two directions at the beginning of each trial (*Direction*(*0*) was set to + *x* or − *x* with equal probability). Herein, *Direction*(*k*) can be − *x* or + *x*, which implies bias in the movement direction. The parameter *prob*(*k*) indicates the probability of moving in *Direction*(*k*). The target agent selected the − *x*/+ *x* direction with equal probability on each time step until it met the foragers. Therefore, the parameter *prob* was set to 0.50 for the target ant (*prob*(*0*) = 0.50), whereas *prob* was set to 0.80 for foraging agents (*prob*(*k*) = 0.80 for *k* > 0). The amount of pheromones on each cell was set to 1 at the beginning of each trial (*pheromone*(*x*, *y*) = 1) and the pheromone evaporation rate *q* was set to 0.99.

The model descriptions are explained using submodels. A Submodel: *the interaction between the target agent and foragers* causes differences between two rules (the reverse-rule model and the alignment-rule model).

#### Submodels

##### Submodel: the interaction between the target agent and foragers

The parameters *Direction*(*0*) and *prob*(*0*) were replaced with new ones whenever the following events occurred.

In the reverse-rule model, for any agent *k* (*k* > 0),
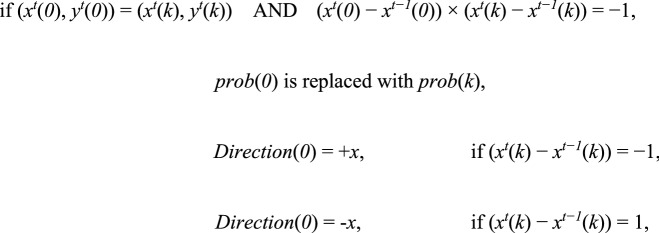


Herein, (*x*^*t*^(*k*), *y*^*t*^(*k*)) indicates the *x–y*-coordinate for the agent *k* at time *t*. Furthermore, (*x*^*t*^(*0*), *y*^*t*^(*0*)) = (*x*^*t*^(*k*), *y*^*t*^(*k*)) means that the target agent and the agent *k* occupy the same cell at time *t* while (*x*^*t*^(*0*) − *x*^*t−1*^(*0*)) × (*x*^*t*^(*k*) − *x*^*t−1*^(*k*)) =  − 1 indicates that the target agent meets the agent *k* came from the opposite direction. The target agent replaces *Direction*(*0*) with an opposite direction from the forager *k* (see Extended data Fig. S5B).

In the alignment-rule model, for any agent *k* (*k* > 0),
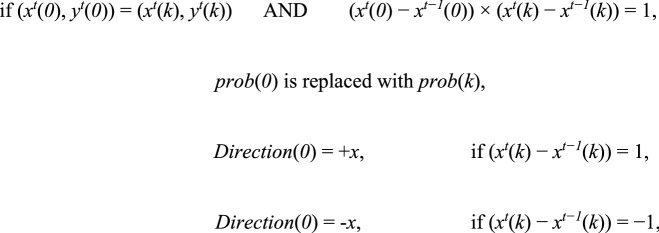


(*x*^*t*^(*0*) − *x*^*t−1*^(*0*)) × (*x*^*t*^(*k*) − *x*^*t−1*^(*k*)) = 1 indicates that the target agent meets the agent *k* came from the same direction. The target agent replaces *Direction*(*0*) with a same direction with the forager *k* (See Extended Data Fig. S5B).

In the reverse-rule model, these events are driven from the experimental observations of real ants. Target ants appear to move against the trail and seem to move straight by contacting those other nestmates that come from the opposite direction. Also, target ants seem to select the reverse goal even if physical contact with ant nestmates does not occur immediately after entering the bridge. So, regarding parameter replacements, we did not consider the position at which the target agent met another agent. Note that foraging agents did not change these parameters until the end of each trial. Further, *Direction*(*0*) can be replaced with − *x* from + *x* and vice versa whenever the target agent meets foragers that come from the opposite direction.

In the alignment-rule model, the target agent tends to move in the same direction with other agents. This is contrary to the experimental observations of real ants.

##### Submodel: position updating

For all *k* agents (*k* = 0–*N*), the movement direction and position updates are shown as follows (Extended Data Fig. S5C);
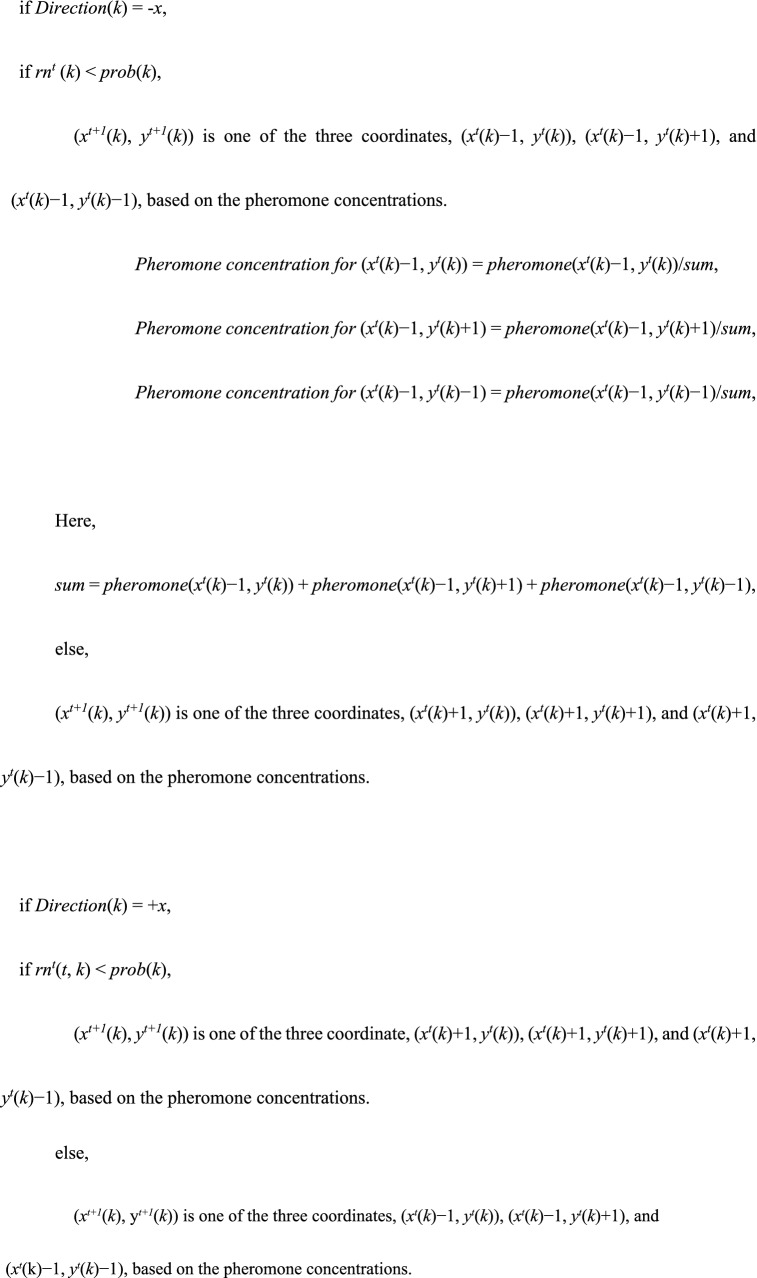


Here, *rn*^*t*^(*k*) indicates a random number. Thus, *rn*^*t*^(*k*) $\in$ [0.00, 1.00].

*Prob*(0) for the target agent is initially set to 0.50. Therefore, the target agent selects one direction from the two (− *x* and + *x*) on each time step randomly before the condition described in submodels—*the interaction between the target agent and foragers* is satisfied. On the other hand, foraging agents select − *x* direction with a high probability (= *Prob*(*k*)) on each time step. After selecting one direction from two (− *x* and + *x*), agents scan three cells in the direction of movement. Using pheromone concentrations on those three cells, they update their positions.

If agents reach coordinates satisfying their *y*-coordinate = 4 or 0, those agents update their position by selecting not three but two coordinates since they are located on the edges of the bridge.

##### Submodel: pheromone updating

Foraging agents (*k* > 0) deposited pheromones on the current cell when leaving that cell.
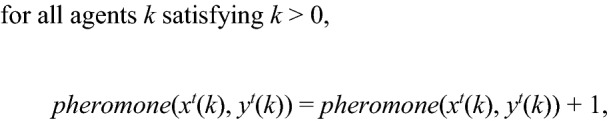


Then, pheromones are evaporated using the evaporation rate *q*.
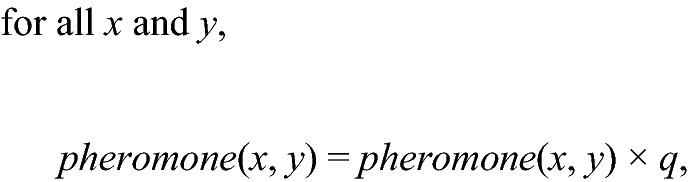


For each time iteration, these submodels operated in the following order.

STEP 1: *The interaction between the target agent and foragers.*

STEP 2: *Position updating.*

STEP 3: *Pheromone updating.*

#### Analysis

To check the accuracy of our model, we counted which goal side the target agent entered the goal line from using the reverse-rule model by setting *N* = 9. If the target agent passed the goal line at *x*-coordinate =  − 30 (30), we considered that it reached the normal (reverse) goal. Note that trials in which the target agent never reached any goal lines by *t* = 500 were excluded from this analysis. Furthermore, to investigate the adaptability of the reverse run mechanism, we examined the time until the target agent reached the goal lines using the reverse-rule model and the alignment-rule model. Herein, we set two different conditions with respect to the number of foraging agents (*N* = 4 and 9).

## Results

### Ant experiments

Seven colonies were used in the main experiment, comparision experiments and a single path experiment to avoid colony dependence (Extended Data Tables S2, S3, and S4). The flow rates of the trails in the main experiment were approximate between 1 ant/1 s and 1 ant/4 s when individual target ants entered the main bridge.

**Figure 3:**
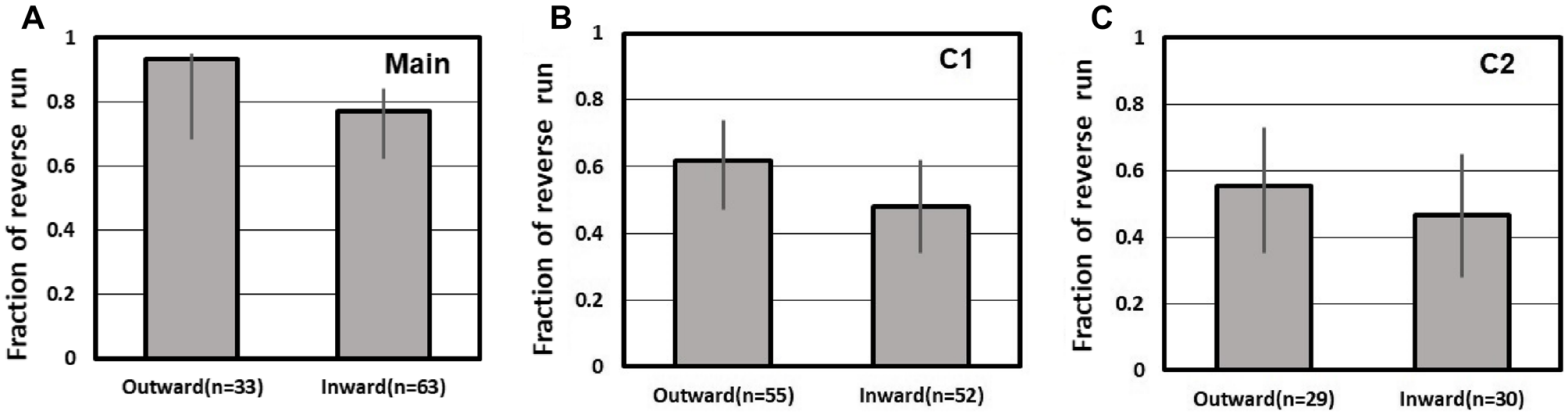
Fraction of the reverse run (goal) against the normal run (goal). (**A**) Main experiment. (**B**) Comparison experiment 1. (**C**) Comparison experiment 2. The error bars indicate the confidence intervals. The term *n* indicates the number of trials.

In the main experiment, in 28 out of 33 trials on the outward path, the direction to the nest (reverse runs) was chosen. Contrastingly, in 47 out of 63 trials on the inward path, the direction to the feeding site was selected. A binomial test was performed for both the outward and inward runs, and it was found that the target ants ran against the flow of the ant trail more frequently (Fig. 3, outward: 28 trials (reverse runs) vs. 5 trials (normal runs), *p*-value < 0.001; inward: 47 trials (reverse runs) vs. 16 trials (normal runs), *p*-value < 0.001).

Next, in the comparison experiment 1, we found that 34 trials out of 55 trials on the outward path were reverse runs. On the inward path, 25 trials out of 52 trials were reverse runs. These results indicate that in comparison experiment 1, when there are no other ants and only pheromone information is available, the trail is selected randomly (Fig. 3, outward: 34 trials (reverse runs) vs. 21 trials (normal runs), *p*-value = 0.10; inward: 25 trials (reverse runs) vs. 27 trials (normal runs), *p*-value = 0.90).

Finally, in the comparison experiment 2, there were 16 reverse runs out of 29 trials on the outward path and 14 reverse runs out of 30 trials on the inward path. A binomial test was conducted (Fig. 3, outward: 16 trials (reverse runs) vs. 13 trials (normal runs), *p*-value = 0.70; inward: 14 trials (reverse runs) vs. 16 trials (normal runs), *p*-value = 0.90). In the comparison experiment 2, there was no information about other ants or pheromones, indicating that both outward and inward paths were selected randomly.

**Figure 4:**
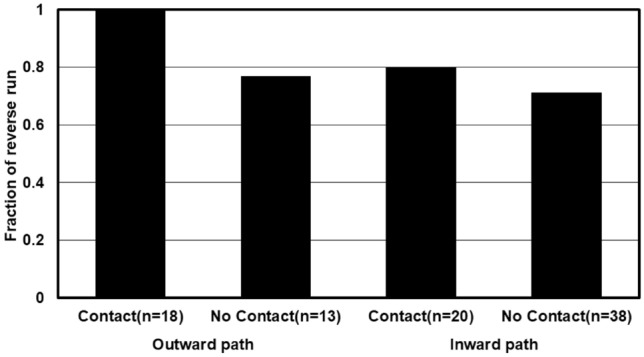
Influence of body contact with another foraging ant on the target ants immediately after joining the bridge. Based on the trials of the main experiment, the presence or absence of contact was examined in the outward and inward paths, and the fraction of the reverse run (goal) against the normal run (goal) with respect to contact and no contact was displayed respectively. The term *n* indicates the number of trials.

Moreover, we conducted an analysis focusing on the contact using the data from the main experiment and observed that target ants interacted with another ant if their bodies came in direct contact with each other. As shown in Fig. 4, where χ^2^ test was performed to evaluate significant differences regarding the ratio between two groups, we found that contact with other ants within 2 cm at the T-junction had no effect on the goal choice (outward: 18 reverse runs out of 18 trials (contact) vs. 10 reverse runs out of 13 trials (no contact), χ^2^ = 2.33, *df* = 1, *p-value* = 0.13; inward: 16 reverse runs out of 20 trials (contact) vs. 27 reverse runs out of 38 trials (no contact), χ^2^ = 0.18, *df* = 1, *p*-value = 0.70). Note that this result does not mean that target ants move against the ant flows without any body-contacts. We excluded trials where target ants never contacted with their nestmates (although it was extremely rare). This result suggests that contact with other ants immediately after entering the T-junction had no effect on the goal choice.

Finally, we would like to comment on a single path experiment. In this preliminary experiment, we found that target ants chose the goal side randomly (See Extended Data File S1).

### Simulation

Per our findings, the target agent seemed to reach the reverse goal more often than the normal goal by setting *N* = 9 (0.79 (reverse) vs. 0.21 (normal)). This tendency disappears when no direct physical contacts between the target agent and other foragers are allowed by setting *N* = 0, i.e., only one target agent was set on the simulation field (0.51 (reverse) vs. 0.49 (normal)). Furthermore, when we set only one target agent (*N* = 0) and no pheromones were deposited on the field, pheromone information did not appear to serve as a key factor affecting the target agent to reach the reverse goal (0.54 (reverse) vs. 0.46 (normal)).

**Figure 5:**
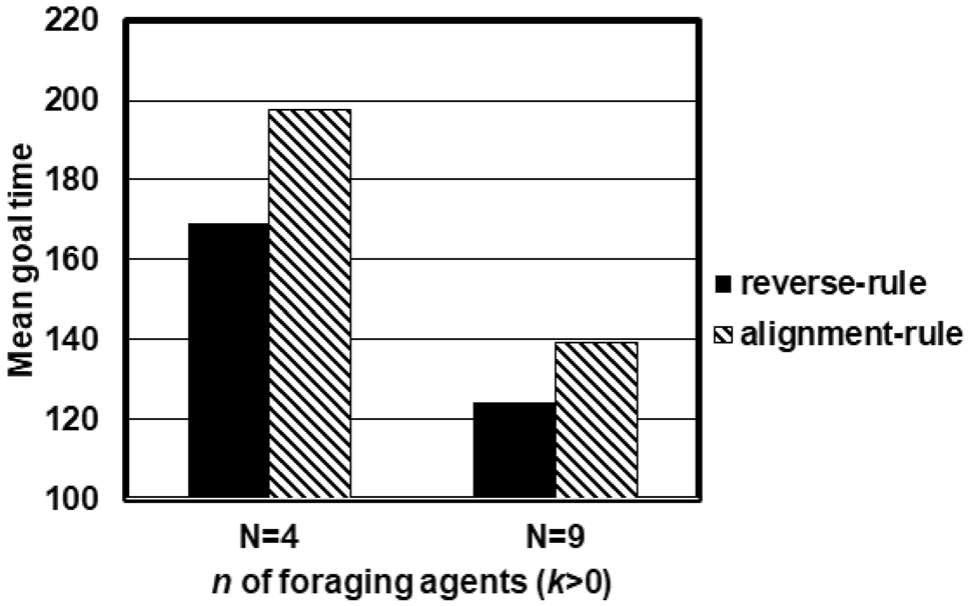
Mean goal time obtained from 1000 trials for each *N* category and each model category. *N* indicates the number of foraging (nontarget) agents.

As shown in Fig. 5, the target agent in the reverse-rule model appeared to reach goal lines in a shorter amount of time than the target agent in the alignment-rule model (Here, *N* means the number of foraging agents. *N* = 4, 169.0 ± 131.4 (reverse-rule) vs. 197.8 ± 144.3 (alignment-rule), *N* = 9, 124.4 ± 108.8 (reverse-rule) vs. 139.3 ± 121.2 (alignment-rule), respectively). Please note that we did not consider which goal the target agent passed. Interestingly, the target agent in the alignment-rule model seemed to reach the normal goal, but not so often compared with results of the reverse-rule model (*N* = 4, 0.40 (reverse) vs. 0.60 (normal), *N* = 9, 0.36 (reverse) vs. 0.64 (normal)) because the target agent who goes ahead with other agents may change its movement direction by following the movement of those other agents, which results in backing and forth. These findings suggest that ants may reach a certain location effectively by contacting other ants and by going against the trail.

We also replaced *prob* = 0.80 with 0.9 for foraging agents (*k* > 0) using *N* = 9 and found that the target agent in the reverse-rule model still reached goal lines in a shorter amount of time than the target agent in the alignment-rule model without being affected by parameter replacement (132.7 ± 132.0 (reverse-rule) vs. 147.1 ± 138.5 (alignment-rule), respectively).

## Discussion

In this study, we used an apparatus with two paths to separate outward flows from inward flows. Target ants that had been isolated from the nest in advance were allowed to join the established ant trails. The results of these experiments suggested that the direction of movement of the target ants may be determined by the presence of other ants when they reach the trails. Considering the results of this study, we concluded that the target ant may make decisions and move in the opposite direction in ant trails in the presence of both pheromones as well as other ants. In previous studies, complementary use of several information seemed to determine the actions of *L. niger* foragers^8,12,13,25^. Thus, we confirm that pheromones and other ants served as information for the target ants in determining the direction to move forward. We also propose that the direct contact immediately after entering the main bridge is not important for the target ants, suggesting which position the direct contact occurs is not important for target ants. When returning to the nest, foraging ants deposit pheromones on their inward path. On the other hand, experienced ants deposit foraging pheromones on both the inward and outward paths^21^. Therefore, it is possible for the target ants to deduce that there is a feeding site by moving in the opposite direction of ant flows and update their direction. Further investigations such as forcing target ants to join a trail only of inexperienced ants will clarify these possibilities.

Our findings were contrary to our expectations as we expected that the target ants would move in the same direction as their nestmates^17^. In some collective behaviors of animals, the alignment rule can be a key point in explaining the emergent property of those living systems^26,27^. For example, birds get closer to each other and progressively align their headings. Notably, we found that target ants chose the goal side randomly when we used a single path in the main apparatus. Then, why do the target ants move in the opposite direction from the direction of other ants rather than moving in the same direction as them? This could perhaps be related to the continuous updating of their actions^5,28^. We think that contact with other foraging ants may provide reliable directional cues to the target ants considering that pheromones do not offer them any directional information when they join a trail. By doing so, the target ants will not get lost and trace a pheromone trail more effectively, thereby helping them reach the desired place.

Our findings do not suggest that ants conduct information transfer regarding food positions when contacting each other^16^. Rather, our findings imply that the target ants are allowed to reinforce forward movement when contacting their nestmates. In fact, we also developed a multiagent model and found that the target agents reached a goal more often when moving against an artificial ant flow than when moving along an artificial ant flow. These findings suggest that naïve ants may adopt adaptive decision-making when they encounter an ant on a pheromone trail as they do not know in which direction the destination, such as food location or their nest, is located^8,13,14^.

Although desynchronizing the flow of outward and inward ants has been observed in laboratory experiments on the garden ant *L. niger*^29^, in natural conditions, the outward path is not separated from the inward path of ant trails. Having said that, our findings indicate an interesting notion—ants may maintain their travel direction along a pheromone trail as long as they continuously encounter nestmates, thereby leading them to a certain destination. This mechanism will help a naïve forager to reliably reach a certain location when it cannot detect any pheromone gradients. At a glance, physical contact between foraging ants is likely to interfere with the forward motion of ant traffic. Considering our results however, it will have an adverse effect on ant flow. This flow property of ant traffic implies that masses of foraging ants may be beyond an aggregation of solid particles. Notably, masses of some ant species do not always behave like a solid, which is dependent on the situation^30,31^. Individual ants can behave adaptively according to the environment condition and modify their actions via an interaction with nestmates. Thus, individuals of living particles influence the macro-pattern of the ensemble^32,33^. To this end, our findings imply and predict a behavioral significance for foraging ants: they may determine the direction to move forward by continuously encountering nestmates on a pheromone trail after leaving the nest. This implication will be particularly true for naïve ants and will be linked to a self-organizing process that contribute to rapid flow and efficient transportation on ant traffic. Further experiments are warranted to evaluate whether it is possible for naïve ants to present reverse runs when they join a pheromone trail not at a right angle, join a pheromone trail not alone but with ant nestmates, or join a pheromone trail not on the one-way flow system.

## Supplementary Information

Supplementary Information.

## Data Availability

All data generated or analyzed during this study are included in this published article and its supplementary information files.
