# Supplementary material for: Ant Lasius niger joining one-way trails go against the flow
Source: Sci Rep. 2022 Feb 11;12:2361. doi: 10.1038/s41598-022-05879-4 (PMC8837658; doi:10.1038/s41598-022-05879-4)
Supplement: Supplementary file 1 — Supplementary Information. [file 41598_2022_5879_MOESM1_ESM.docx]

**EXTENDED DATA**

**Methods: A single path experiment**

In this study, we also investigated bi-directional traffic behavior using an apparatus that had a single path from the nest to the feed (Extended Data Figure 4, 30×2×15 cm). In the same way as in the main experiment of the two-path apparatus, individual target ants entered the apparatus path using the confluence device after the establishment of ant traffic on a pheromone trail. Two joining points were set on the center of the single path (the right (R) and left (L) sides seen from the nest). We set the goal lines at 15 cm from the center of the path. We checked which side (the nest side or the feeder side) a target ant passed the goal line. If all ants located within 10 cm of the target ant moved in one direction (to the feed or to the nest), this trial was excluded from the analysis. We defined a (nontarget) ant’s flow in the same way as in the main experiment of the two-path apparatus (See Extended Data Figure 3).

**Results: A single path experiment**

Four colonies were used (#1~4), and 67 trials (L: 35 trials, R: 32 trials) were conducted. We found that the individual target ants randomly selected goals when they were allowed to join a bi-directional ant traffic (32 (15 (L) and 17 (R)) out of 67 target ants goaled to the nest, *X-squared* = 0.35478, *df* = 1, *p-value* = 0.55).

Extended Data Table 1. Colony information. Date refers to the collection date. Place refers to the place where the colonies were collected from.

| Date | Place | Colony Number # |
| --- | --- | --- |
| 2020/5/2 | University | 1 |
| 2020/5/2 | University | 2 |
| 2020/6/10 | Park | 3 |
| 2020/6/10 | Park | 4 |
| 2020/7/19 | Park | 5 |
| 2020/8/8 | Park | 6 |
| 2020/9/1 | Park | 7 |

Extended Data Table 2. The experimental results (main experiment) for each colony with respect to the goal. Colony # refers to the colony number. The other numbers indicate the total number of trials counted as the normal goal or the reverse goal for each category (LL, LR, RR, and RL).

| Date of the experiment | Colony # | LL |  | LR |  | RR |  | RL |  |
| --- | --- | --- | --- | --- | --- | --- | --- | --- | --- |
|  |  | normal | reverse | normal | reverse | reverse | normal | reverse | normal |
| 2020/6/24 | 3 | 1 | 7 |  |  | 5 | 0 |  |  |
| 2020/7/22 | 5 | 2 | 5 |  |  | 2 | 0 |  |  |
| 2020/7/31 | 5 | 0 | 3 | 0 | 4 | 5 | 0 | 3 | 0 |
| 2020/8/11 | 6 | 1 | 3 | 1 | 6 | 1 | 0 | 1 | 3 |
| 2020/8/16 | 6 |  |  |  |  | 7 | 4 | 7 | 3 |
| 2020/9/22 | 7 |  |  |  |  | 8 | 1 | 8 | 5 |
| Total |  | 4 | 18 | 1 | 10 | 28 | 5 | 19 | 11 |

Extended Data Table 3. The experimental results (comparison experiment 1) for each colony with respect to the goal. Colony # refers to the colony number. The other numbers indicate the total number of trials counted as the normal goal or the reverse goal for each category (LL, LR, RR, and RL).

| Date of the experiment | Colony # | LL |  | LR |  | RR |  | RL |  |
| --- | --- | --- | --- | --- | --- | --- | --- | --- | --- |
|  |  | normal | reverse | normal | reverse | reverse | normal | reverse | normal |
| 2020/8/13 | 6 | 1 | 2 | 1 | 1 | 1 | 1 | 1 | 1 |
| 2020/8/28 | 6 | 2 | 5 | 3 | 3 | 4 | 1 | 1 | 4 |
| 2020/9/1 | 6 | 3 | 6 | 4 | 7 | 4 | 6 | 5 | 5 |
| 2020/9/9 | 7 | 4 | 5 | 3 | 5 | 2 | 3 | 7 | 6 |
| Total |  | 10 | 18 | 11 | 16 | 11 | 11 | 14 | 16 |

Extended Data Table 4. The experimental results (comparison experiment 2) for each colony with respect to the goal. Colony # refers to the colony number. The other numbers indicate the total number of trials counted as the normal goal or the reverse goal for each category (LL, LR, RR, and RL).

| Date of the experiment | Colony # | LL |  | LR |  | RR |  | RL |  |
| --- | --- | --- | --- | --- | --- | --- | --- | --- | --- |
|  |  | normal | reverse | normal | reverse | reverse | normal | reverse | normal |
| 2020/8/28 | 6 | 2 | 3 | 3 | 1 | 2 | 2 | 2 | 4 |
| 2020/8/31 | 6 | 4 | 6 | 4 | 6 | 4 | 6 | 6 | 4 |
| Total |  | 6 | 9 | 7 | 7 | 6 | 8 | 8 | 8 |


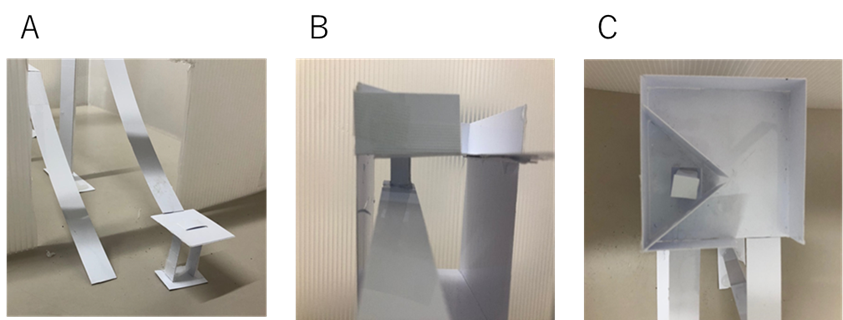


Extended Data Figure 1. Structure used to prevent reverse flow. A. The slope of the experimental apparatus connected to the nest. The outward path (left side) can be entered smoothly by foraging ants from the nest, but the inward path (right side) cannot be entered easily by ants from the nest due to the rat-guard structure. B. The connection from the outward path to the feeding site. A rectangular hole was made in the feeding site, and a rectangular rod was inserted through the hole as a vertical structure. C. The feeding site seen from above. Immediately after the ants were allowed to climb the vertical structure, they must pass a small gap to enter the feeding site. A wall was erected to narrow the gap, thereby preventing them from entering the feeding site from the outward path.


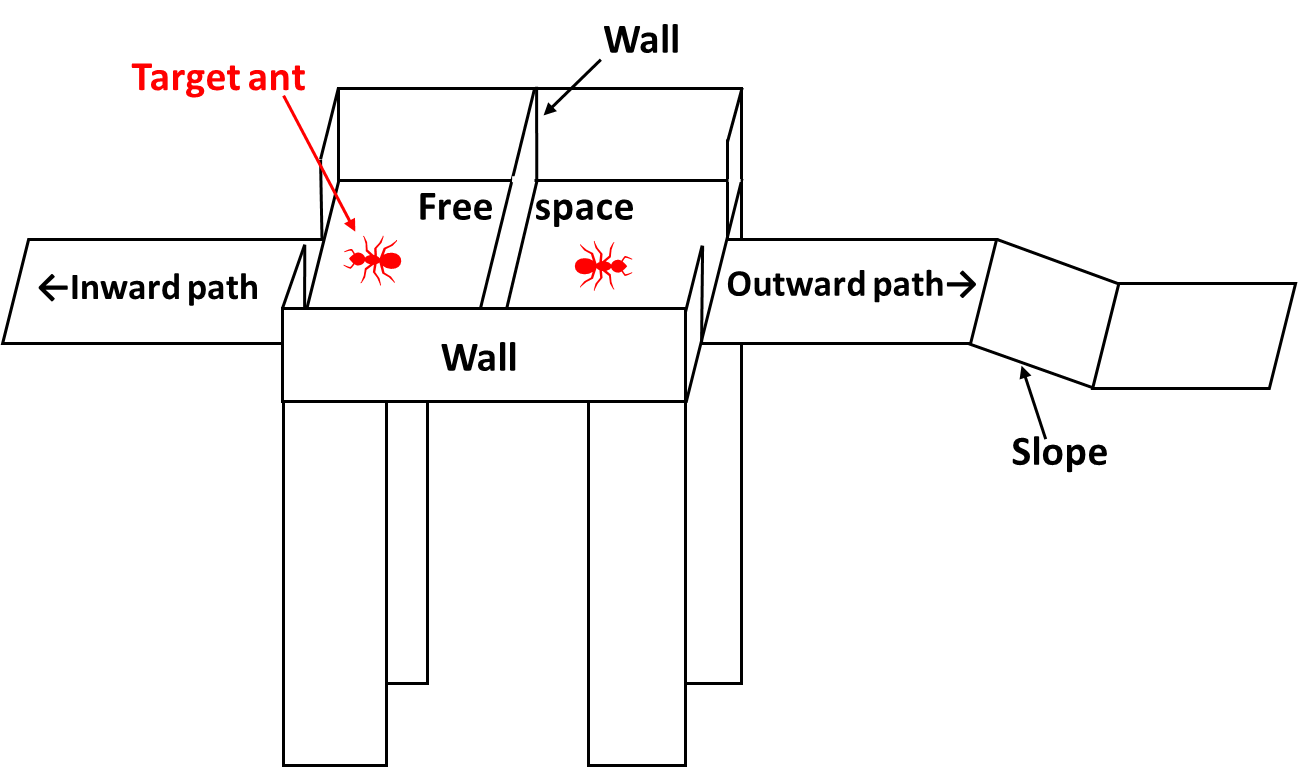


Extended Data Figure 2. Confluence device. The left side part of this apparatus is for the ants to join the inward path, and the right side part is for the ants to join the outward path. Free space was provided for the ants to settle down, preventing them from falling and ensuring natural merging. The reason for the longer confluence path on the outward part is that the heights of the outward and inward bridges of the main apparatus were different, and a slope was built only on the outward confluence path. A wall of 5 cm was built in the free space.


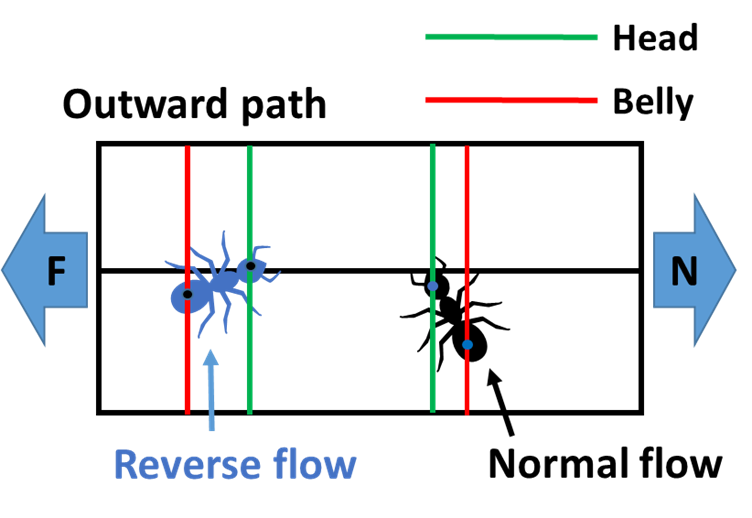


Extended Data Figure 3. Definition of reverse and normal flow of other ants. There are green and red lines perpendicular to a parallel straight line connecting the feeding site and the nest. The green line was fixed at the center of the ant’s head and the red line at the center of the ant’s belly. On the outward path, we defined a normal flow when the green line was closer to the feeding site than the red line, and a reverse flow when the green line was closer to the nest than the red line. The inward path was defined in the same way.


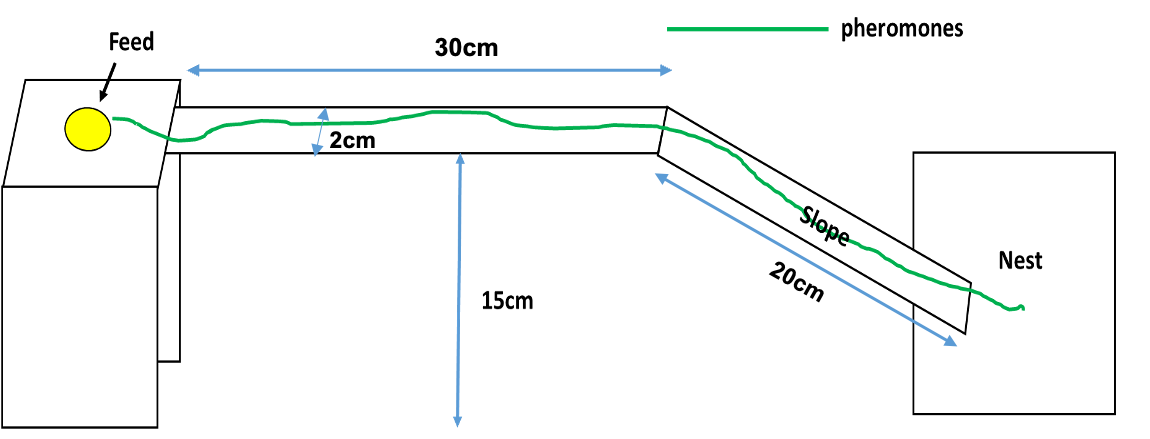


Extended Data Figure 4. The main apparatus used in the single path experiment. Nests are connected to the experimental apparatus by a slope. In this experiment, there is an ant traffic from the nest to the feed and from the feed to the nest on a pheromone trail.


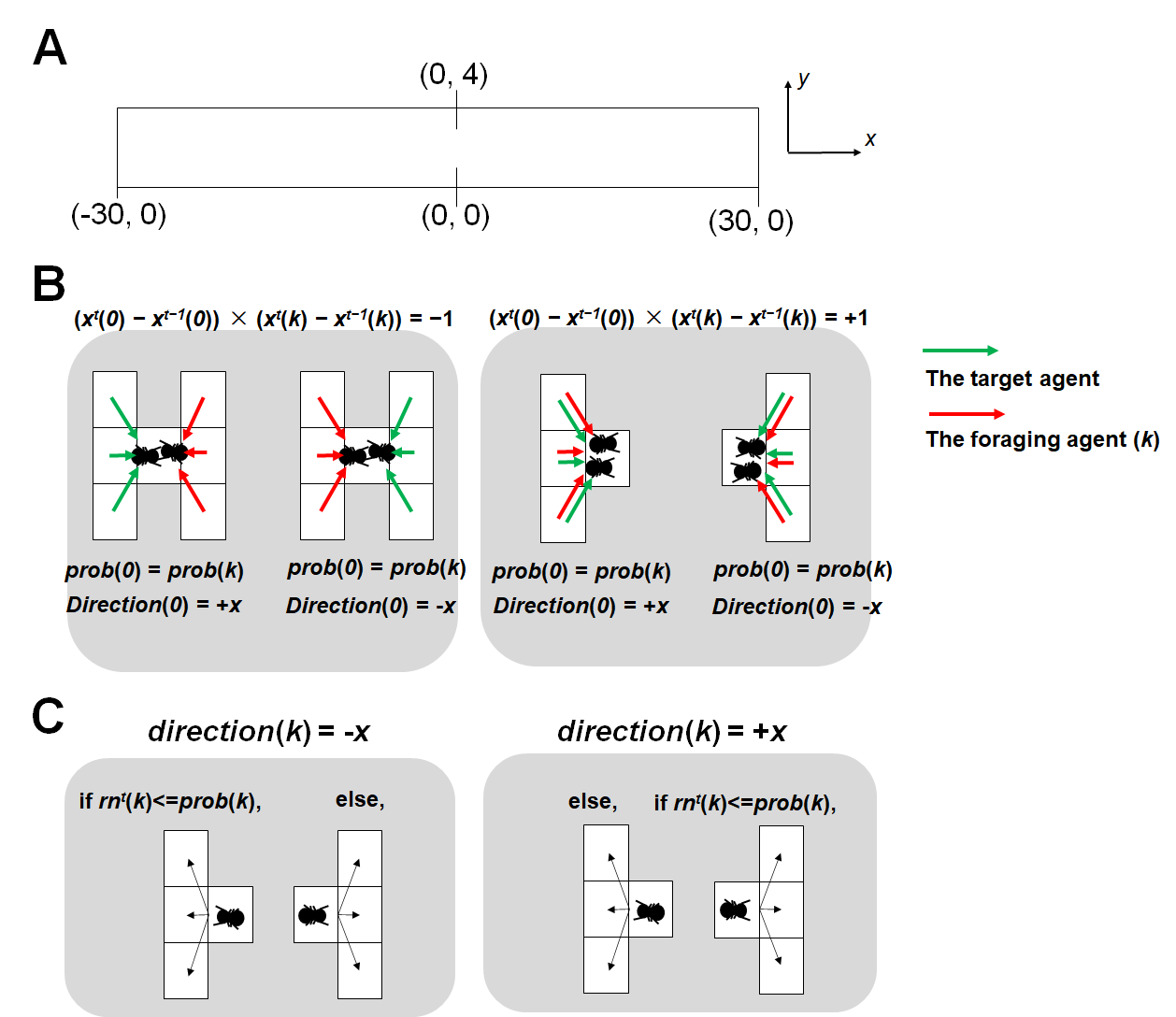


Extended Data Figure 5. Schematic illustrations for the experimental bridge and submodels (“the interaction between the target agent and foragers” and “position updating”). A. Experimental bridge used for simulation experiments. The target agent was set on the coordinate (0, 2). The normal goal line was set on the *x*-coordinate = −30. The reverse goal line was set on the *x*-coordinate = 30. Foraging agents moved in *left* (-*x* direction). B. Illustrations for the interaction between the target agent and foragers. White squares indicate cells. The target agent and the agent *k* occupy the same cell. The reverse-rule model (left figure) and the alignment-rule model (right figure) are shown. Green and red arrows indicate movements from possible previous cells to the current cell for the target agent and the agent *k* respectively. Here, *k* = 1, 2, …, *N*. C. Illustrations for position updating. White squares indicate cells. Agents choose one cell from three by scanning pheromone concentrations on those cells. Here, *k* = 0, 1, …, *N*.
